# High Seroprevalence of Severe Fever with Thrombocytopenia Syndrome Virus Infection among the Dog Population in Thailand

**DOI:** 10.3390/v15122403

**Published:** 2023-12-11

**Authors:** Keita Ishijima, Thanmaporn Phichitraslip, Nattakarn Naimon, Preeyaporn Ploypichai, Benyapa Kriebkajon, Torntun Chinarak, Jirasin Sridaphan, Anamika Kritiyakan, Noppadol Prasertsincharoen, Sathaporn Jittapalapong, Kanate Tangcham, Worawut Rerkamnuaychoke, Yudai Kuroda, Masakatsu Taira, Kango Tatemoto, Eunsil Park, Milagros Virhuez-Mendoza, Yusuke Inoue, Michiko Harada, Tsukasa Yamamoto, Ayano Nishino, Aya Matsuu, Ken Maeda

**Affiliations:** 1 Department of Veterinary Science, National Institute of Infectious Diseases (NIID), Tokyo 162-8640, Japan; keishi@niid.go.jp (K.I.); ykuroda@niid.go.jp (Y.K.); taira@niid.go.jp (M.T.); ktatemoto@niid.go.jp (K.T.); peunsil@niid.go.jp (E.P.); mvirhuez@niid.go.jp (M.V.-M.); yinoue@niid.go.jp (Y.I.); hmichiko@niid.go.jp (M.H.); tyama@niid.go.jp (T.Y.); tantan55@niid.go.jp (A.N.); matsuu@niid.go.jp (A.M.); 2 Faculty of Veterinary Technology, Kasetsart University, Bangkok 10900, Thailand; cvttpp@ku.ac.th (T.P.); nattakarn.n@ku.th (N.N.); preeyaporn.ploy@ku.th (P.P.); benyapa.krie@ku.th (B.K.); torntun.chi@ku.th (T.C.); jirasin.sri@ku.th (J.S.); anamika.k@ku.ac.th (A.K.); sbcndp@ku.ac.th (N.P.); fvetspj@ku.ac.th (S.J.); 3 Office of Veterinary Public Health, Department of Health, Bangkok 10400, Thailand; job_1184@hotmail.com; 4 Faculty of Veterinary Medicine, Rajamangala University of Technology Tawan-ok, Chonburi 20110, Thailand; worawut148@yahoo.com; 5 Joint Graduate School of Veterinary Medicine, Yamaguchi University, Yamaguchi 753-8515, Japan

**Keywords:** severe fever with thrombocytopenia syndrome, companion animal, dogs, zoonosis, RT-PCR, ELISA, Thailand

## Abstract

Severe fever with thrombocytopenia syndrome (SFTS) is an emerging tick-borne zoonotic disease caused by the SFTS virus (SFTSV). In Thailand, three human cases of SFTS were reported in 2019 and 2020, but there was no report of SFTSV infection in animals. Our study revealed that at least 16.6% of dogs in Thailand were seropositive for SFTSV infection, and the SFTSV-positive dogs were found in several districts in Thailand. Additionally, more than 70% of the serum samples collected at one shelter possessed virus-neutralization antibodies against SFTSV and the near-complete genome sequences of the SFTSV were determined from one dog in the shelter. The dog SFTSV was genetically close to those from Thailand and Chinese patients and belonged to genotype J3. These results indicated that SFTSV has already spread among animals in Thailand.

## 1. Introduction

Severe fever with thrombocytopenia syndrome (SFTS) is an emerging tick-borne zoonotic disease caused by the SFTSV virus (SFTSV), which is formally named Dabie bandavirus belonging to genus *Bandavirus*, family *Phenuiviridae*, order *Bunyavirales* according to the classification of the International Committee on Taxonomy of Viruses. The case fatality rate in humans is high, although the rates differ between countries: the rate is reported to be 27% in Japan [1], 21.6% in Korea [2], and 5.2% to 16.2% in China [3,4]. The detection of SFTSV in humans has been reported in China, Japan, Korea, Taiwan, Vietnam, Myanmar, and Pakistan, and the virus is presumed to be spreading mainly in East and Southeast Asia [5,6,7,8,9,10,11,12,13]. In Thailand, the first human case of SFTS was reported in 2019 [13]. Rattanakomol et al. reported one human case of SFTS in 2019 and two cases of SFTS in 2020 [14].

SFTSV infects various mammals, including sheep, cattle, deer, wild boars, dogs, cats, cheetahs, raccoons, mink, yellow weasels, rodents, shrews, and hedgehogs [15,16,17,18,19,20,21,22]. In our previous summary of SFTSV infection in Japan until the end of September 2022, we found 560 cases of SFTS in cats and 36 cases of SFTS in dogs [23]. In several cases of human SFTS infection, including veterinarians and pet owners, SFTSV was directly transmitted from cats or dogs with SFTS, and not from ticks [12,24,25]. In endemic Asian countries, SFTS is a serious zoonotic disease that should be controlled.

The detection of the SFTSV viral RNA in dogs was first reported in 2013 in China [26]. SFTSV was detected in 6.6% and 4% of the dogs in Laizhou Province and Penglai Province, respectively. Han et al. reported 14 cases of dogs infected with SFTSV between April 2019 and December 2020 at animal hospitals in Korea [27]. These dogs showed some symptoms that were similar to those seen in cases of SFTS in humans and cats, but there were no deaths [28,29]. In Japan, three of seven dogs (43%) infected with SFTSV died between April 2017 and November 2019. The dogs with SFTS exhibited decreased activity, fever, leukopenia, and thrombocytopenia [22]. Similar to that in humans, the case fatality rate in dogs may vary between countries.

Seroepidemiological studies on SFTSV infection in dogs have been performed in Japan, Korea, and China; the seropositivity of anti-SFTSV immunoglobulin (Ig) G in dogs ranged from 0.53% to 1.9% in Japan [30,31], 7.4% to 37.9% in China [26,32], and 13.9% to 21.4% in Korea [33,34].

In the present study, we used an enzyme-linked immunosorbent assay (ELISA) to detect anti-SFTSV antibodies in serum samples from dogs in Thailand, and positivity for anti-SFTSV antibodies was subsequently confirmed by a virus-neutralization (VN) test. Additionally, we determined the genomic sequences of the SFTSV detected from one dog in Sattahip, where the seroprevalence of SFTSV infection among dogs was high.

## 2. Materials and Methods

### 2.1. Animal Samples

All of the dog serum samples used in this study were collected in Thailand between April 2021 and April 2022. Samples were collected from 458 dogs in 16 districts of six provinces (Supplementary Figure S1). This study was approved by Kasetsart University under permission ID number ACKU65-VTN-005. Blood samples were centrifuged at 822× *g* for 5 min, then the supernatant was collected and stored at −20 °C until the detection of antibodies and/or viral RNA analysis.

### 2.2. ELISA

For the screening of dogs with anti-SFTSV antibodies, ELISA was performed as described previously [22]. The SFTSV HB-29 strain [5] was kindly provided by Dr. Dexin Li and Dr. Mifang Liang of the National Institute for Viral Disease Control and Prevention, Chinese Center for Disease Control and Prevention. The optical density (OD) values of the SFTSV-infected cell lysates were normalized by subtracting the OD values of the mock-infected cell lysates. The cut-off OD value for IgG was determined to be 0.129, which was previously calculated using an ELISA and VN test for detecting anti-SFTSV IgG in dogs in Japan; the specificity and sensitivity were both 92.3% at this cut-off value (our unpublished data).

### 2.3. VN Test

For the confirmation of SFTSV positivity, the 50% focus reduction neutralization test (FRNT_50_) was determined using the sera from the ELISA-positive dogs as described previously [22]. The FRNT_50_ was determined as the reciprocal of the highest dilution when the number of foci was <50% of the number of plaques in wells without serum. Titers over 1:10 were taken to indicate positivity for antibodies against SFTSV.

### 2.4. RT-PCR

For the detection of the SFTSV viral RNA, reverse transcription-polymerase chain reaction (RT-PCR) was performed. Viral RNA extraction was performed using a QIAamp Viral RNA Mini Kit (QIAGEN, Hilden, Germany) according to the manufacturer’s protocol. Sixty microliters of RNA were extracted from 140 µL of serum. RT-PCR for the detection of SFTSV RNA was performed using two sets of previously reported primers: SFTSV-S2-200s and SFTSV-S2-360a, and SFTSV-S7F and SFTSV-S7R [22]. The reaction conditions were the same as in the previous report. The RT-PCR products were separated by electrophoresis on a 2% agarose gel, and the sizes were confirmed after staining with Gel-Red.

### 2.5. Sequence Analysis

The RNA samples in which the SFTSV viral RNA was detected were used for amplification of the viral RNA. RT-PCR for viral genome amplification was performed as described previously [22]. The RT-PCR products were purified using a FastGene Gel/PCR Extraction Kit (FastGene, Tokyo, Japan), and analyzed by direct sequencing. The determined nucleotide sequences were deposited in the DNA Data Bank of Japan (DDBJ) database (large (L) segment: LC733203; medium (M) segment: LC733204; and small (S) segment: LC733205). Homology of nucleotide sequences was analyzed using the Basic Local Alignment Search Tool [35] and the Sequence Manipulation Suite [36].

### 2.6. Phylogenetic Analysis

Three FASTA files, including the SFTSV nucleotide sequences of the S, M, and L segments, were prepared. The sequences of Guertu virus [37] were used as an outgroup. Sequence alignments were performed using MAFFT online service [38]. A selection of optimal DNA models and a phylogenetic analyses were performed using IQ-tree v1.6.12 for windows [39] with 1000 ultrafast bootstrap repetitions [40] and the Shimodaira–Hasegawa approximate likelihood ratio test [41].

## 3. Results

### 3.1. Detection of Anti-SFTSV Antibodies from Dogs

The results of the first screening for anti-SFTSV antibodies revealed that 106 dogs (23.1%) were seropositive for anti-SFTSV IgG antibodies (Table 1).

Subsequently, among the 106 positive samples, 98 samples that had a sufficient volume for further testing were used in the FRNT_50_ to confirm the positive results of the ELISA. The results confirmed that 76 of the 98 dog serum samples were positive for SFTSV. The minimum positive ratio for anti-SFTSV antibodies was 16.6% among dogs in Thailand. Surprisingly, the minimum positive ratio among dogs in Sattahip, Chonburi, was very high at 52.6%. We analyzed the data from the SFTSV-positive dogs and found that most SFTSV-positive serum samples were collected at one shelter in Sattahip in February 2022. In this shelter, 50 of 64 dogs (minimum positive ratio: 78%) were found to be positive for anti-SFTSV antibodies by the FRNT_50_.

### 3.2. Detection of SFTSV from One Dog

**Figure 1:**
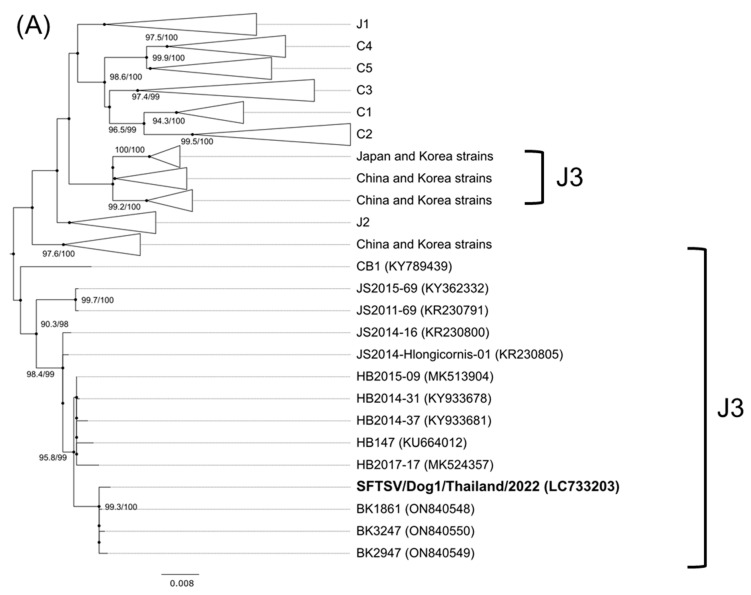

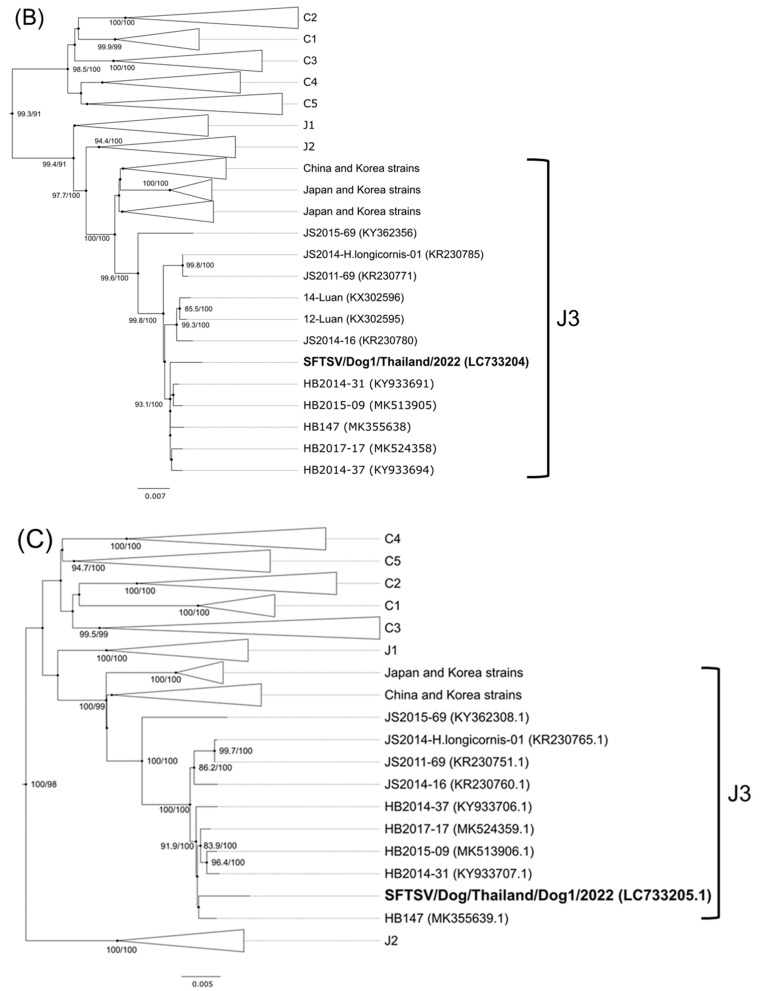
Phylogenetic analysis by the maximum likelihood method based on the nucleotide sequences of the small (**A**), medium (**B**), and large (**C**) segments. The Guertu virus that was used as an outgroup is not shown. The black circle indicates the nodes. The bold text indicates the virus strain detected in this study. The number at each branch indicates the Shimodaira–Hasegawa approximate likelihood ratio test (SH-aLRT) value (%)/ultrafast bootstrap (UFBoot) value (%). These values are shown when SH-aLRT value is ≥80% and UFBoot value is ≥95%.

Since mass infection of SFTSV appeared to have occurred at this shelter around February 2022, we performed RT-PCR to detect the SFTSV viral RNA in 57 serum samples collected from dogs in Sattahip in February 2022. Seven samples could not be tested due to an insufficient sample volume. We detected the SFTSV viral RNA in one dog serum sample, and subsequently determined the complete protein-coding sequences of the S, M, and L segments. The virus was named SFTSV/Dog/Thailand/Dog1/2022. The nucleotide sequences of the segments were the most closely related to the sequences of the following SFTSV strains from Thailand and China: the S segment showed a 99.7% similarity in identity to that of Thailand SFTSV BK1861; the M segment showed a 99.0% similarity in identity to that of Chinese SFTSV HB2014-31; and the L segment showed a 99.2% similarity in identity to that of Chinese SFTSV HB147. Phylogenetic analysis also indicated that this dog SFTSV was closely related to those of genotype J3 [42] (Figure 1).

## 4. Discussion

In 2019, Ongkittikul et al. reported the first case of a human infected with SFTSV in Thailand [13]. Other reports in 2019 and 2020 also indicated that SFTSV has been spreading in Thailand [14]. Our study showed that at least 16.6% of dogs in Thailand were seropositive for SFTSV infection, and the SFTSV-positive dogs were found in several districts (Table 1). These results also indicated that SFTSV has been spreading in many districts in Thailand.

The seroprevalence among dogs in Thailand (16.6%) was more similar to those reported in China and Korea than that reported in Japan [26,28,30,31]. The nucleotide sequences of SFTSV determined in this study are more closely related to the sequences of SFTSV in China than those in Japan. The prevalence of SFTSV infection among the dog population in Thailand may be similar to those in endemic regions of China.

The SFTSV viral RNA was detected in only one sample collected from a healthy dog at the shelter in Sattahip in February 2022. Surprisingly, more than 70% of the serum samples collected at this shelter possessed anti-SFTSV antibodies. However, there was no information on the clinical symptoms, if any, in the dogs at this shelter around February 2022. SFTSV in Thailand might not cause severe disease in dogs. Further investigation is necessary to clarify the pathogenesis of SFTSV in Thailand.

To our knowledge, this is the first report on of all three segments of the SFTSV genome in Thailand. The nucleotide sequence of the S segment of SFTSV detected from the dog in Thailand was closely related to that isolated from patients in Thailand (Figure 1). In addition, the SFTSV sequence was detected in the dog in Sattahip district, Chonburi Province. Two of three SFTS patients in Thailand lived in Chachoengsao Province next to Chonburi Province [14]. It was presumed that SFTSV has been spreading in these areas. The nucleotide sequences of M and L segments of the SFTSV isolated in Thailand were the most similar to those in China. Nonetheless, further information on the genomes of SFTSV strains is required to clarify the origin of the SFTSV in Thailand.

At least three human cases have already been reported in Thailand and many dogs in Thailand possessed anti-SFTSV antibodies, indicating that SFTSV has already spread in Thailand and more human cases will occur in the future, similar to in other Asian countries [5,6,7,8,9,10,11]. Furthermore, Thailand people, especially veterinarians, should keep in mind that SFTSV can directly transmit from SFTS dogs to their owners or veterinarians without tick-bites [12,21]. In addition, all seven cats owned by the first reported SFTS patient in Thailand died in over a week [13], indicating the possibility that direct transmission of SFTSV occurred from the cats to the patient. To assist with prevention of SFTSV infection in Thailand, it is necessary to conduct further surveillance of SFTSV infection in animals.

## Figures and Tables

**Table 1:** Results of the anti-SFTSV IgG ELISA and virus-neutralization test in dogs in Thailand.

Province	District	ELISA (OD > 0.129)		50% Focus Reduction Neutralization Test (≥1:10)		Minimum Positive Ratio (%)
		No. of Examined Dogs	No. of Positive Dogs	No. of Examined Dogs	No. of Positive Dogs	
Prachinburi		17	1	1	0	0.0
Bangkok		143	12	12	6	4.2
Chachoengsao		18	2	2	2	11.1
Samutprakan		81	19	16	10	12.3
Rayong		14	2	2	1	7.1
Chonburi	Mueang Chonburi	27	6	6	3	11.1
	Bang Lamung	56	5	5	3	5.4
	Sattahip	95	56	52	50	52.6
	Pattaya city	7	3	2	1	14.3
Total		458	106	98	76	16.6

## Data Availability

The data shown in this research are available in the article.

## Supplementary Materials

The following supporting information can be downloaded at: https://www.mdpi.com/article/10.3390/v15122403/s1, Figure S1: Map of the provinces in which dog serum samples were collected in this study.
