# Supplementary material for: High Seroprevalence of Severe Fever with Thrombocytopenia Syndrome Virus Infection among the Dog Population in Thailand
Source: Viruses. 2023 Dec 11;15(12):2403. doi: 10.3390/v15122403 (PMC10747823; doi:10.3390/v15122403)
Supplement: Supplementary file 1 [file viruses-15-02403-s001.zip › viruses-2712497-supplementary.pdf]

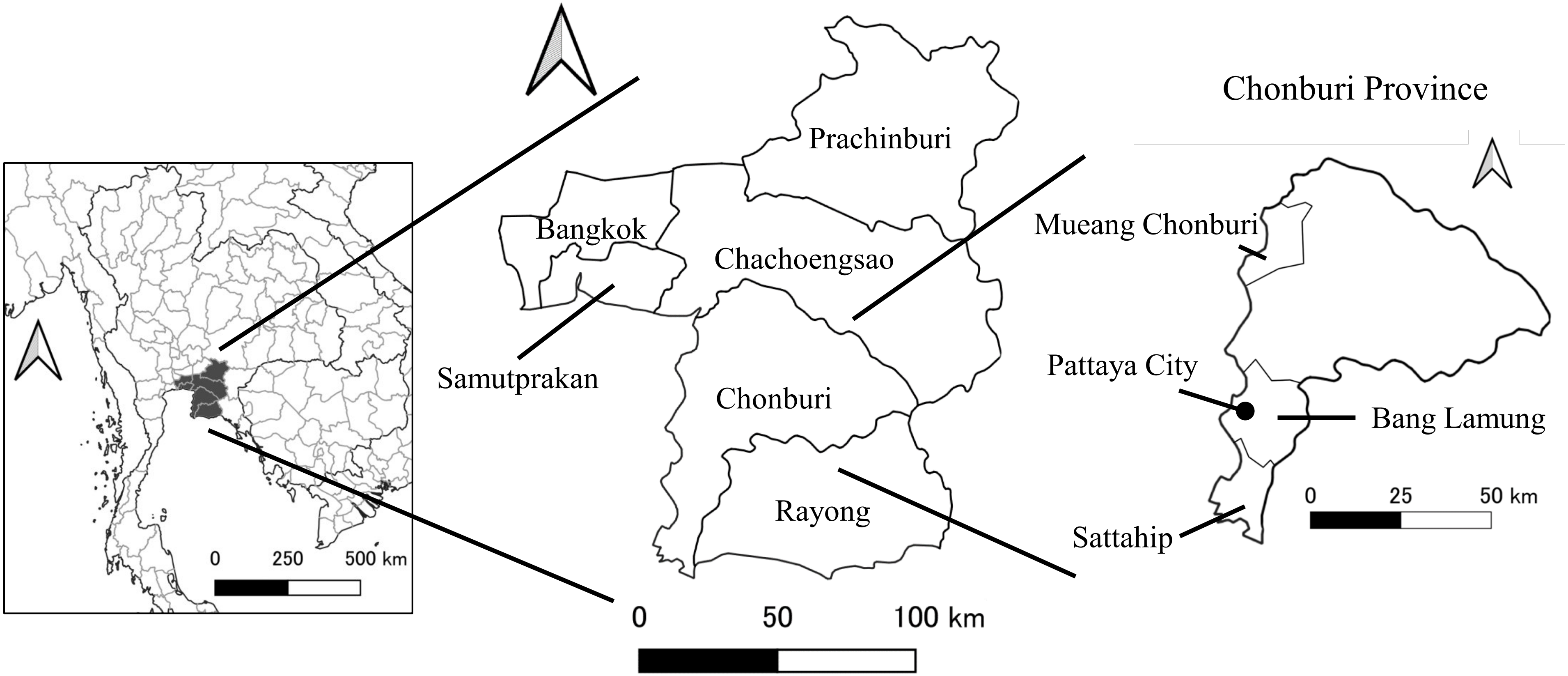

Supplemental Figure S1. Map of the provinces in which dog serum samples were collected in this study. On the left, the gray area indicates the provinces in which we collected dog samples. Each province is labeled on the map in the middle (Prachinburi, Bangkok, Chachoengsao, Samutprakan, Rayong, and Chonburi). On the right, the three districts (Mueang Chonburi, Bang Lamung, and Sattahip) and the city (Pattaya) in Chonburi province, where we collected dog samples for this study, are labeled. Pattaya city is a self-governing municipal area, and not part of the Bang Lamung district.
